# Evaluation of Microsatellites Markers to Discriminate Four Main Taeniid Tapeworms of Dogs

**Published:** 2017

**Authors:** Saeedeh SHAMSADDINI, Mohammad Ali MOHAMMADI, Seyed Reza MIRBADIE, Saeid NASIBI, Sima ROSTAMI, Mansoureh DEHGHANI, Majid FASIHI HARANDI

**Affiliations:** 1. Research Center for Hydatid Disease in Iran, School of Medicine, Kerman University of Medical Sciences, Kerman, Iran; 2. Dept. of Medical Laboratory Sciences, Sirjan School of Medical Sciences, Kerman University of Medical Sciences, Kerman, Iran; 3. Dept. of Basic Medical Sciences, School of Medicine, Shahroud University of Medical Sciences, Shahroud, Iran; 4. Dept. of Research and Technology, Alborz University of Medical Sciences, Karaj, Iran

**Keywords:** *Echinococcus*, *Taenia*, Microsatellite markers, Transmission, EmsB

## Abstract

**Background:**

*Echinococcus granulosus, Taenia multiceps, Taenia ovis* and *Taenia hydatigena* are among the most prevalent taeniid species of dogs. These tapeworms infect ruminant and humans as intermediate hosts and domestic/wild carnivores as the definitive hosts. Molecular tools using hypervariable microsatellite regions might provide more information about parasite variation. Highly variable and specific tools are needed for transmission tracking studies of canine echinococcosis as an essential element for implementation of hydatid control programs.

Suitable microsatellite markers used so far are EmsJ, EmsK, EmsB, EMms1, Egmsca1, Egmsga1, U1 snRNA. The purpose of the present study was to determine the microsatellite variability of EmsB as well as six other microsatellites in major taeniid species infecting dogs in Iran.

**Methods:**

Twenty isolates of each of the four Taeniidae tapeworms were collected from sheep during routine veterinary inspection in Tehran, Alborz and Kerman provinces from October 2010 to May 2011. After DNA extraction, PCR was set up with optimum conditions using specific primers for each individual microsatellite marker. All the PCR products were evaluated by agarose gel electrophoresis. We used SDS-PAGE for evaluating patterns of PCR products in the tapeworms.

**Results:**

*E. granulosus* as well as *Taenia* species could be differentiated based on EmsB microsatellite patterns. The electrophoresis patterns of two taeniid genera were readily distinguishable. EmsB could be specifically used in epidemiological studies of canine echinococcosis.

**Conclusion:**

Different patterns of EmsB proved this microsatellite marker as a reliable tool for epidemiological studies on canine echinococcosis.

## Introduction

Cestodes of the family Taeniidae occur as adult tapeworms in the small intestine of carnivorous definitive hosts such as dogs and are transmitted to specific mammalian intermediate hosts e.g. sheep and goats, where they develop as fluid-filled larvae in the host tissues (1). Different species of Taeniid tapeworms are prevalent in carnivores especially dogs in Iran among them *Echinococcus granulosus, Taenia multiceps, Taenia ovis* and *Taenia hydatigena* are the most prevalent and present major public health and economic losses in the country (2).

Investigating these tapeworm infections in dog’s in particular canine echinococcosis is a major component of any comprehensive hydatid control program (1).

Transmission tracking of the infection in the definitive hosts using molecular tools plays a key role to improve our understanding of the nature and significance of different patterns of disease transmission (3). Because all taeniid eggs are morphologically indistinguishable, molecular discrimination between *E. granulosus* and *Taenia* species is of paramount importance in molecular epidemiological studies of canine echinococcosis (4).

Microsatellites known as simple sequence repeats (SSR) or short tandem repeats (STR) are abundant unique tandem repeats of short (2–6 bp) DNA motifs in eukaryotic genomes which are usually polymorphic in number because of their high abundance, widespread distribution in the genomes of various organisms (5). Due to insufficient variations present in many mitochondrial and nuclear regions for identifying geographical location-based genetic variation and discriminating between individual isolates, the use of highly variable regions such as microsatellites provides more information about the parasite and the spatial and temporal characteristics of its patterns of transmission among different geographical locations.

U1 snRNA gene was the first microsatellite region described in the taeniid species, *E. multilocularis* (6). The marker was shown to be able to differentiate different isolates of *E. multilocularis* from Europe, North America, and Japan. EMms 1 and EMms 2 were later used to study population genetics and discriminate different isolates of *E. multilocularis* from Hokkaido, Japan (7). Different studies have been carried out on a series of microsatellite regions of *Echinococcus* namely EmsJ, EmsK, EmsB, Egmsca1, Egmsga1 (8, 9).

One of the most suitable microsatellite markers used so far is EmsB, a multi-locus, multi-copy genetic marker that provides enough sensitivity to trace *Echinococcus* transmission. EmsB has been shown to be a valuable tool for tracking origin of *E. multilocularis* from different hosts and geographical locations (10). EmsB also demonstrated potential for studies on cystic echinococcosis caused by *E. granulosus* s.l. and other taeniid infections in human and animals. However, our knowledge on the extent of EmsB variation in major taeniid species of dogs, i.e. *E. granulosus, T. multiceps, T. ovis* and *T. hydatigena* needs to be improved.

The purpose of the present study was to determine variability of EmsB and six other microsatellite markers among four major taeniid species of dogs in Iran.

## Materials and Methods

Twenty metacestode isolates of each of the four Taeniidae tape-worms, *E. granulosus* sensu stricto*, T. ovis, T. hydatigena* and *T. multiceps* were collected from sheep at different slaughterhouses in Kerman and Tehran provinces from October 2010 to May 2011. The study design was approved by the KMU Research Ethics Committee No. 93–232. All 80 isolates were characterized by PCR sequencing in previous studies (11–13).

DNA extraction was carried out as described elsewhere (12). Briefly, the protoscoleces/germinal layers were minced, frozen, thawed six times, and then incubated overnight in 200mg tissue lysis buffer and 80mg proteinase K at 56 °C. The remaining steps of DNA extraction were conducted using High Pure PCR Template Preparation Kit (Roche, Mannheim, Germany), according to the manufacturer’s instructions.

Seven primer pairs were used for PCR amplification of the microsatellite markers (Table 1). Each primer set was initially tested for amplification by PCR in a 30 μl reaction mixture containing 1.65 mM MgCl_2_, 1.65 mM MgCl_2_, 0.11 units/μl Amplicons Taq DNA polymerase, 10–40 pmol of each primer and 4m l (50 – 100 ng/ml) of the DNA template. PCR products were electrophoresed in 20 cm long 8–12% polyacrylamide gels and visualized by AgNO_3_ staining. The banding patterns of each set of isolates were compared between and within each species.

**Table 1:** Primer sequences and characteristics of microsatellite loci used in characterization of four major taeniid species in Iran

**Primer**	**Sequence**	**Annealing temp (°C)**	**Reference**
EmsJ,	5_-GAACGCGCTAACCGATTG-3_, 5_-TTAGGAATGGGAAGGTGTCG-3	54	(14)
EmsK	5_-CAGCTCAAAAGAACCCGAAG-3_, 5_-CCAAACTTCCGCTCACTCTG-3_	54	(14)
EmsB	5_-GTGTGGATGAGTGTGCCATC-3_, 5_-CCACCTTCCCTACTGCAATC-3_	60	(14)
EMms1	5_-GGTAGCCAATGCTGTGGTTT-3_, 5_GCGAGGTCACGCAAATGTAT_3	60	(14)
Egmsca1	F:5- CGAAAGTGATGACAAACCAA – 3 R-GCTTGATGGAGATGAGGTCG-3	55	(15)
Egmsga1	F:5-TGACGGCGATGATGAGATAG-3 R:5-CCTTGCCACACGCTACACTG-3	55	(15)
U1 snRNA	59-GACATTGTCGTTGCCATCTCTCCCA-39 59-TTAGAGTCCGTCGCAGGCTTCAAC-39	55	(6)

## Results

**Fig. 1:**
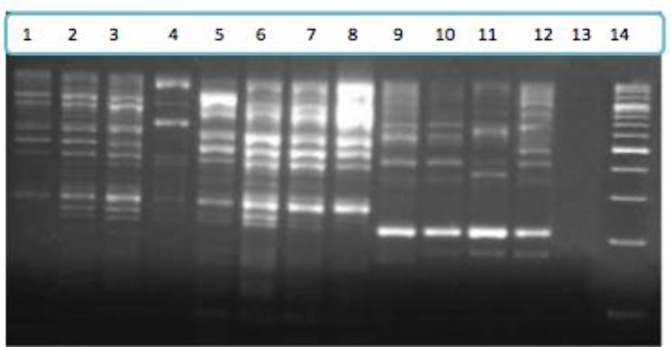
Agarose gel electrophoresis showing the fragments amplified by PCR for EmsB microsatellite region in three *Taenia* species; *Taenia hydatigena* 1–4, *Taenia ovis 5–8, Taenia multiceps 9–12,* negative control 13, DNA size marker 14.

EmsB fragments were amplified in all four species of taeniids. PCR amplification of EmsB microsatellite region showed different banding patterns in each species. No bands were produced in negative control reactions. Each individual *Taenia* species showed a specific pattern for EmsB on agarose gel electrophoresis (Fig. 1). The patterns were readily distinguishable from *E. granulosus* sensu stricto*.* In addition, isolates within each individual species demonstrated uniform bands in different reactions.

**Fig. 2:**
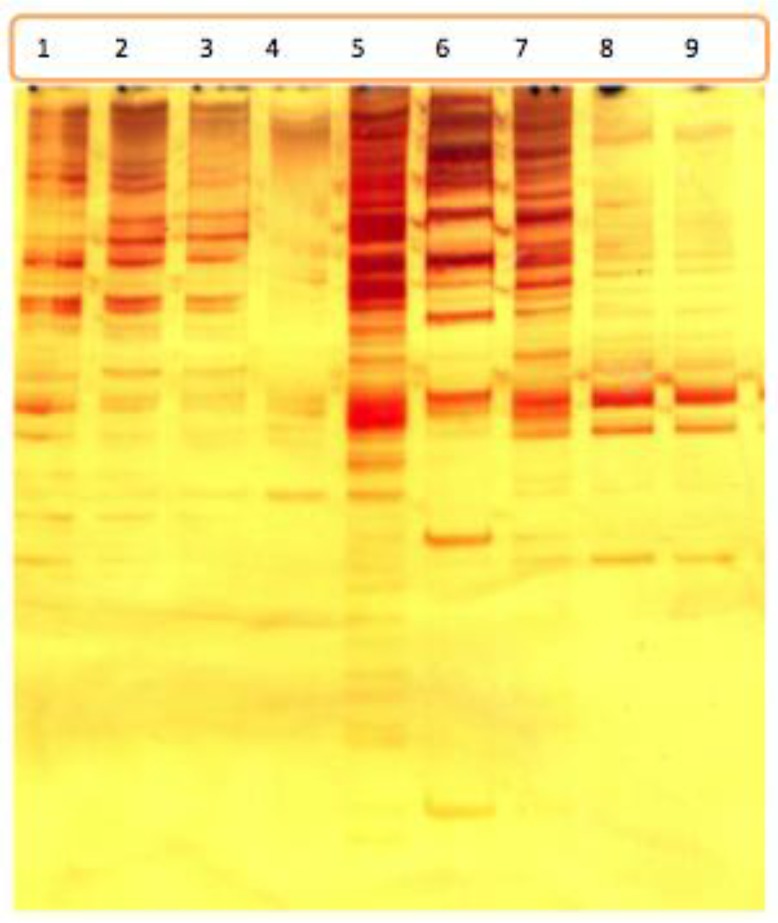
Polyacryl Amide Gel Electrophoresis (PAGE) showing distinctive banding pattern for EmsB in four taeniid species; *Taenia multiceps* 1–2*, Taenia ovis* 3–4*, Taenia hydatigena* 5 and 7, DNA size marker 6, *Echinococcus granulosus* sensu stricto 8–9.

These findings were further confirmed by Polyacryl amide gel electrophoresis (Fig. 2). Different PAGE patterns were demonstrated for each of EmsJ, EmsK, EMms1, U1 snRNA, Egmsca, Egmsga loci, however, fragments that are more variable were demonstrated in EmsB and the number of bands within each pattern was higher in EmsB region in all four taeniid tapeworms indicating the suitability of this microsatellite locus for molecular epidemiological studies.

## Discussion

Comparing microsatellite markers in taeniid tapeworms EmsB shows substantial variability among *Echinococcus* species. Tandemly repeat multi-locus nature of EmsB makes this marker a suitable target for genetic polymorphism studies (9, 16, 17). In addition, EmsB is a useful tool for transmission tracking studies in canine echinococcosis. However, the significance and extent of variation in microsatellite markers in other taeniid species have not been fully understood. No data on EmsB variability are available for major *Taenia* species in dogs.

In the present study, specific patterns of EmsB were demonstrated in three major *Taenia* species in dogs. Distinctive EmsB patterns for each of major dog *Taenia* species is crucially important for the applicability of this tool for transmission tracking studies of canine echinococcosis as well as other Taeniids. EmsB provided more variable fragments than other microsatellite markers investigated in the present study. The difference of the patterns among four species reflects the unique fingerprints of each of microsatellite regions in *Taenia* species. EmsB is a suitable microsatellite tool to detect *Echinococcus* fingerprints in mixed infections with closely related *Taenia* species. As the biotic potential of *E. granulosus*, *T. hydatigena* and *T. ovis* have shown to be well-correlated (18), tracking transmission of these species in the same host could provide important information for modeling the disease and selecting control strategies. Using this tool, field molecular epidemiological studies in endemic regions could provide valuable information on the dynamics of transmission in the region (19).

High degree of variability and discriminatory power of EmsB were demonstrated in 127 isolates of E. granulosus from six endemic countries of Asia, Africa, Europe and South America (20). This microsatellite proved as a useful locus for assessing *Echinococcus* populations at a focal / regional scale. Dogs also play an important role in the epidemiology of alveolar echinococcosis in several endemic areas (9, 21–23). Repetitive sequence and electropherogram fragments in *E. granulosus* were more variable than that of *E. multilocularis* suggesting the applicability of this tool to differentiate between the two species. This is especially important in the areas where cystic and alveolar echinococcosis are co-existed and dogs are infected by the both species.

Obviously, microsatellite regions, in particular, EmsB are potentially applicable for surveillance of CE as part of hydatid control programs. However, widespread field studies are required to validate this tool as a reliable technique for transmission dynamic analyses on cystic echinococcosis.

## Conclusion

The optimum variability of EmsB microsatellite region among major Taeniidae tape-worms of dogs allows us to set appropriate tools to discriminate different canine taenids. Different patterns of EmsB proved this microsatellite marker as a reliable tool for epidemiological studies on canine echinococcosis.
